# Role of Brain-Derived Neurotrophic Factor in Beneficial Effects of Repetitive Transcranial Magnetic Stimulation for Upper Limb Hemiparesis after Stroke

**DOI:** 10.1371/journal.pone.0152241

**Published:** 2016-03-23

**Authors:** Masachika Niimi, Kenji Hashimoto, Wataru Kakuda, Satoshi Miyano, Ryo Momosaki, Tamaki Ishima, Masahiro Abo

**Affiliations:** 1 Department of Rehabilitation Medicine, The Jikei University School of Medicine, Tokyo, Japan; 2 Division of Clinical Neuroscience, Chiba University Center for Forensic Mental Health, Chiba, Japan; 3 Department of Rehabilitation Medicine, Tokyo General Hospital, Tokyo, Japan; Rutgers University, UNITED STATES

## Abstract

**Background:**

Repetitive transcranial magnetic stimulation (rTMS) can improve upper limb hemiparesis after stroke but the mechanism underlying its efficacy remains elusive. rTMS seems to alter brain-derived neurotrophic factor (BDNF) and such effect is influenced by BDNF gene polymorphism.

**Objectives:**

To investigate the molecular effects of rTMS on serum levels of BDNF, its precursor proBDNF and matrix metalloproteinase-9 (MMP-9) in poststroke patients with upper limb hemiparesis.

**Methods:**

Poststroke patients with upper limb hemiparesis were studied. Sixty-two patients underwent rehabilitation plus rTMS combination therapy and 33 patients underwent rehabilitation monotherapy without rTMS for 14 days at our hospital. One Hz rTMS was applied over the motor representation of the first dorsal interosseous muscle on the non-lesional hemisphere. Fugl-Meyer Assessment and Wolf Motor Function (WMFT) were used to evaluate motor function on the affected upper limb before and after intervention. Blood samples were collected for analysis of BDNF polymorphism and measurement of BDNF, proBDNF and MMP-9 levels.

**Results:**

Two-week combination therapy increased BDNF and MMP-9 serum levels, but not serum proBDNF. Serum BDNF and MMP-9 levels did not correlate with motor function improvement, though baseline serum proBDNF levels correlated negatively and significantly with improvement in WMFT (ρ = -0.422, p = 0.002). The outcome of rTMS therapy was not altered by *BDNF* gene polymorphism.

**Conclusions:**

The combination therapy of rehabilitation plus low-frequency rTMS seems to improve motor function in the affected limb, by activating BDNF processing. BDNF and its precursor proBDNF could be potentially suitable biomarkers for poststroke motor recovery.

## Introduction

Stroke is the leading cause of death and the main cause of long-term disability worldwide. Hemiparesis is a common disability in patients with stroke. Previous studies indicated that repetitive transcranial magnetic stimulation (rTMS) can improve poststroke hemiparesis [1,2]. Although rTMS is considered to modulate neuronal plasticity by stimulating cortical excitability, the precise mechanism underlying the beneficial effects of rTMS remains obscure [2,3].

Brain-derived neurotrophic factor (BDNF) plays an important role in neuronal plasticity and in the pathophysiology of various brain disorders [4–9]. Accumulating evidence suggests that BDNF mediates, at least in part, the therapeutic benefits of rTMS. It is reported that rTMS increases blood BDNF (mature BDNF + proBDNF) levels in patients with depression [10,11]. Furthermore, daily 5-Hz rTMS for 5 days significantly increased serum levels of BDNF (mature BDNF + proBDNF) in healthy human subjects, resulting in activation of BDNF-TrkB signaling [12]. Moreover, val66met polymorphism of the *BDNF* gene negatively influences the effect of rTMS on poststroke upper limb hemiparesis [13]. These findings suggest that the observed changes in peripheral blood are due to rTMS-induced modulation of BDNF-TrkB signaling in the brain [12].

ProBDNF, the precursor protein of the mature form BDNF, is converted to BDNF by extracellular proteases, such as matrix metalloproteinase-9 (MMP-9). In this regard, the mature BDNF and proBDNF play important physiological functions by eliciting opposite effects via TrkB and p75^NTR^, respectively [7,8,14]. Mature BDNF could be involved in increased brain excitability, while proBDNF seems to play a role in reducing brain excitability [14]. Considering the high levels of both proBDNF and mature BDNF in the human serum [15] and their putative opposing functions, it is clinically and scientifically interesting to measure the individual serum levels of mature BDNF and proBDNF in human subjects [7,8,16–18]. One previous study indicated that serum levels of mature BDNF, but not proBDNF, were significantly lower in patients with depression than those of healthy control subjects [19]. The same study also showed significant correlations between serum MMP-9 levels and the severity of depression, quality of life, and social function scores in depressed patients [19]. To our knowledge, however, there were no studies that assessed serum levels of mature BDNF, proBDNF, and MMP-9 in stroke patients before and after rTMS.

The present study was designed to determine the molecular effects of rTMS on serum levels of mature BDNF, proBDNF and MMP-9 in poststroke patients with upper limb hemiparesis, and the relationship between serum biomarkers and functional evaluation of upper limb hemiparesis in patients after stroke.

## Materials and Methods

### Subjects

The study subjects were inpatients admitted to Tokyo General Hospital for rehabilitation therapy for hemiparesis after stroke during the period between February 2012 and March 2014. The inclusion criteria were: 1) Age at intervention of 30–90 years. 2) Time after onset of stroke >1 month. 3) Mild or moderate upper limb hemiparesis (able to flex fingers of the affected upper limb). 4) At a probable plateau state with regard to recovery of upper limb hemiparesis as determined by serial evaluation after the onset of stroke [no increase in the Fugl–Meyer Assessment (FMA) score during the latest two-weeks]. 5) No cognitive deficits (Mini Mental State Examination (MMSE) >23 and if with aphasia, Raven’s Colored Progressive Matrices (RCPM) > average score for the same age—2SD). 6) No history of convulsions. 7) No intracranial metal or cardiac pacemaker. The exclusion criteria were: 1) Unacceptable quality of blood samples collected from the patients (hemolysis, insufficient volume and melting of frozen samples), and 2) missing values of clinical evaluation before and/or after intervention.

The study was approved by the ethics committees of The Jikei University School of Medicine and Tokyo General Hospital and the Biomedical Research Ethics Committee of the Graduate School of Medicine at Chiba University. A signed informed consent about participation in this study and rTMS treatment was obtained from each patient.

### Application of rTMS

Low-frequency rTMS inhibits cortical excitability in the stimulated region [20], whereas high-frequency rTMS facilitates cortical excitability [21]. Based on these properties, many studies have reported that low-frequency rTMS should be applied over the primary motor area of the non-lesional hemisphere while high-frequency rTMS should be applied over the primary motor area of the lesional hemisphere in order to improve poststroke upper limb hemiparesis [1]. This is because enhancement of cortical excitability of the lesional hemisphere facilitates motor recovery in stroke patients [22] and the direct effect of high-frequency rTMS or the indirect effect of low-frequency rTMS through a reduction of interhemispheric inhibition towards the lesional hemisphere from the non-lesional hemisphere is expected.

Low-frequency rTMS was used in the present study. For this purpose, a 70-mm figure-8 coil and MagPro R30 stimulator (MagVenture Company, Farum, Denmark) were used for application of rTMS. According to the safety recommendations and hospital protocol [1, 23], we applied 1-Hz rTMS over the motor area that represents the first dorsal interosseous (FDI) muscle on the nonlesional hemisphere. The optimal site of stimulation was defined as the location where the largest motor evoked potentials (MEPs) in the FDI muscle of the unaffected upper limb were elicited on electromyography. The motor threshold (MT) of the FDI muscle of the unaffected upper limb was defined as the lowest intensity of stimulation that could activate MEPs of the FDI muscle. The intensity of stimulation was subsequently set at 90% of the measured MT of the FDI muscle. Each session consisted of 1200 pulses and two sessions were conducted per day. Each patient underwent a total 22 treatment sessions delivered on a daily basis except for holidays.

### Rehabilitation therapy

In both groups, patients received rehabilitation comprising 60-min training in the morning and 60-min training in the afternoon, provided by a physiotherapist, every day over a period of two weeks. Rehabilitation mainly consisted of shaping techniques and repetitive task practice designed to use intensively the affected upper limb. The shaping techniques included reaching forward to move a cup from one place to another, wiping the surface of the table with a towel, picking up a hairbrush and combing hair, writing letters with a pencil, drawing pictures with a pen, handling chopsticks to pick up small objects, folding an umbrella and other activities based on activity of daily living. The repetitive task practice typically included turning over cards, squeezing clay, gripping a small ball, and pinching small coins. Although each 60-min training time usually included 30 minutes of shaping techniques and 30 minutes of repetitive task practice, the proportion of training time was modified depending on improvement in motor function of the affected upper limb, if necessary. In patients who received rTMS, each 60-min training was scheduled to start soon after the application of rTMS.

### Clinical evaluation

The MMSE was evaluated before the treatment. RCPM was used instead of MMSE in patients with aphasia to evaluate cognitive function. The FMA and Wolf Motor Function Test (WMFT) were used to evaluate motor function in the affected upper limb before and after treatment. The RCPM consists of 36 visual multiple-choice tests and does not require verbal responses, so it is used to evaluate cognition in the aphasic patient [24]. The FMA used for assessment of upper limb motor function includes 33 items [25], and each item is rated on a three-point ordinal scale, with a maximum motor performance score of 66 points. The WMFT consists of 15 functional timed tasks and the performance time of each task is measured and summed as the total time [26].

### Blood samples

Blood samples were taken between 8:30 am and 9:00 am after breakfast at 7:00 am at Tokyo General Hospital (Tokyo, Japan). Blood samples were collected twice; before and after treatment. Blood samples before treatment were collected for the purpose of analysis of the *BDNF* Val66Met gene polymorphism and measurement of serum biomarkers. After 14 days of the first blood sampling, blood samples were collected after treatment for measurement of serum biomarkers. The obtained samples were anonymized and immediately stored at -20°C. Within one week of collection, the stored samples were transported under freezing condition to the laboratory of Chiba University Center for Forensic Mental Health and stored at -80°C until subjected to analysis.

### Analysis of BDNF gene polymorphism

Genomic DNA was extracted from blood samples, using the DNeasy Blood & Tissue Kit (Qiagen, CA). *BDNF* 196 A/G polymorphism was assayed, using the protocol described previously [27,28]. Polymerase chain reaction (PCR) and the PCR-based restriction fragment length polymorphism (RFLP) assay were performed to genotype the DNA sequence variants of the *BDNF* gene. PCR was carried out in a total volume of 25 μl with 1 unit of Ex Taq DNA polymerase (Takara Bio, Otsu, Japan) in the reaction mixture. The primer sequences for analysis of val66met (196G>A) (GENBANK: AF411339; at position 95422) in exon XIIIA (position 95206–98892) were forward: 5’-GGTGAGAAGAGTGATGACCA-3’ (position 95214–95233) and reverse: 5’-GCCAGCCAATTCTCTTTTTG-3’ (position 95892–95911). The former contains the first met and the second thr (MT), while the latter contains 223lys, 224lys, 225arg, 226ile, 227gly and 228trp (KKRIGW). The amplification conditions were initiated at 94°C for 4 min, followed by 32 cycles consisting of denaturation at 94°C for 30 sec, annealing at 58°C for 30 sec and extension at 72°C for 30 sec, with a final extension step of 7 min at 72°C. The PCR products were digested at 37°C with restriction enzyme *PmaC*I (Takara Shuzo, Kyoto, Japan) for analysis of 196G>A (val66met) in exon XIIIA, followed by 2% agarose gel- electrophoresis with ethidium bromide staining.

### Measurement of serum levels of proBDNF, BDNF and MMP-9

Serum levels of proBDNF, mature BDNF, and MMP-9 were measured using the human proBDNF Rapid ELISA Kit (Biosensis, Thebarton, SA, Australia), the human BDNF ELISA Kit (Aviscera Bioscience, Santa Clara, CA), and the human MMP-9 ELISA Kit (R&D Systems, Minneapolis, MN), respectively. To minimize assay variance, serum levels of proBDNF, mature BDNF, and MMP-9 were measured in each subject in a single day. All measurements were performed in duplicates and the protocols were performed according to the instructions provided by the manufacturers. The optical density of each well was measured using an automated microplate reader (Emax; Molecular Devices, Sunnyvale, CA).

### Statistical analysis

All statistical analyses were performed using SPSS version 21.0 (IBM, Somers, NY). The Student’s *t*-test was used for comparison of normally distributed parameters and the Mann-Whitney test was used for comparison of parameters that showed skewed distribution pattern. The chi-squared test was used for comparison of categorical data. Data were analyzed using two-way analysis of variance (ANOVA). Correlations between clinical variables and serum biomarkers were carried out using Spearman’s correlation. Data of WMFT, proBDNF, and MMP-9, which showed skewed distribution patterns, were log-transformed to allow meaningful statistical analysis. A *P* value of less than 0.05 was considered to denote statistical significance.

## Results

The rTMS plus rehabilitation group included 62 patients, while rehabilitation without rTMS group included 33 patients. The baseline clinical characteristics of all patients are summarized in Table 1. There were no differences in the clinical features of patients of the two groups.

**Table 1:** Comparison of clinical characteristics at baseline.

	Rehabilitation with rTMS group (n = 62)	Rehabilitation without rTMS group (n = 33)	p value
Age (years), mean±SD	62.3±11.0	66.2±10.8	0.097
Female, n (%)	21 (33.9)	16 (48.5)	0.189
Subtype of stroke			0.174
Hemorrhage, n (%)	34 (54.8)	14 (42.4)	
Infarction, n (%)	28 (45.2)	19 (57.6)	
Stroke lesion			0.392
Cortical, n (%)	8 (12.9)	6 (18.2)	
Subcortical, n (%)	49 (79.0)	22 (66.7)	
Brain stem, n (%)	5 (8.1)	5 (15.2)	
FMA, mean±SD	50.2±12.4	52.9±12.7	0.307
WMFT log performance time, mean±SD	2.02±1.45	1.70±1.29	0.303
MMSE, median [IQR]	29.0, [28.0–30.0]	29.0, [27.0–30.0]	0.616

SD: Standard Deviation; FMA: Fugl-Meyer Assessment; WMFT: Wolf Motor Function Test; MMSE: Mini-Mental State Examination; IQR: Interquartile range.

### Improvement of motor function in affected upper limb after 2-week rTMS

**Fig 1:**
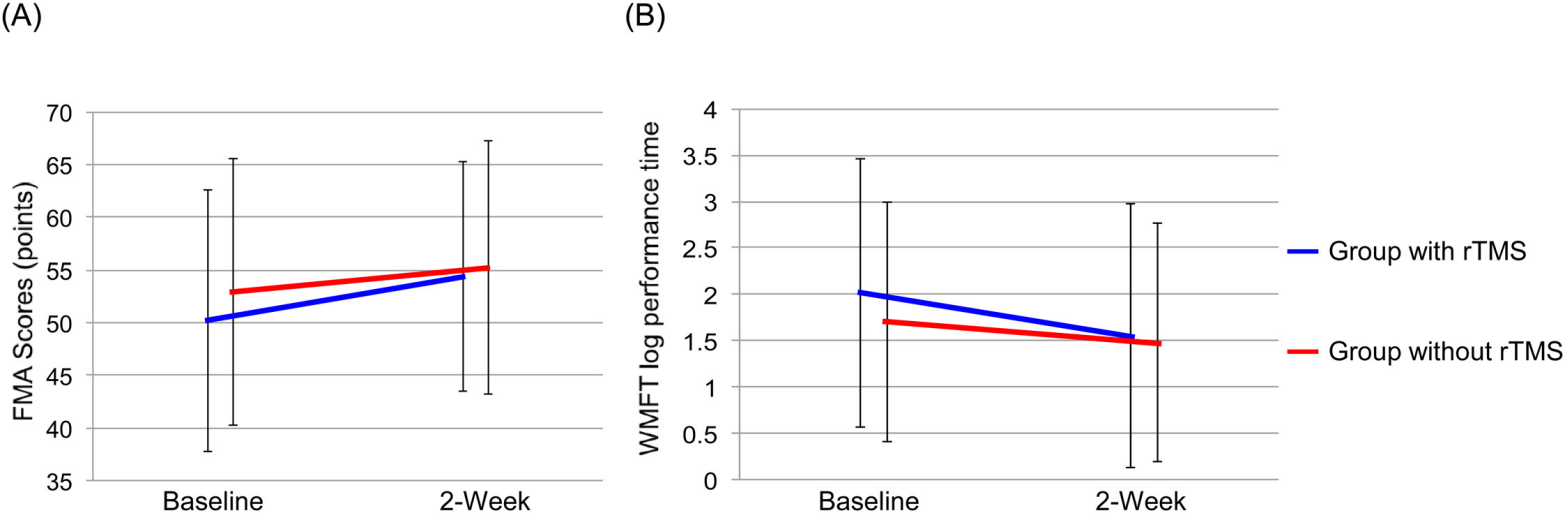
Effect of rehabilitation with and without 2-week rTMS on FMA and WMFT log performance time. (A) FMA: There was a significant interaction between group and time (F = 7.4, df = 1,93, p = 0.008). (B) WMFT log performance time: There was a significant interaction between group and time (F = 4.6, df = 1,93, p = 0.035).

None of the patients who received rTMS treatment showed any adverse effects. Two-way repeated measures ANOVA of FMA data showed a significant time effect (group: F = 0.5, df = 1,93, p = 0.476, time: F = 87.6, df = 1,93, p<0.001) and a significant group-by-time interaction (F = 7.4, df = 1,93, p = 0.008) (Fig 1A). The results showed that the increase in FMA was significantly higher in the rTMS plus rehabilitation group than the rehabilitation only group.

Two-way repeated measures ANOVA of WMFT log performance time showed a significant time effect (group: F = 0.4, df = 1,93, p = 0.507, time: F = 39.7, df = 1,93, p<0.001) and a significant group-by-time interaction (F = 4.6, df = 1,93, p = 0.035) (Fig 1B). The results showed that the decrease in WMFT log performance time was significantly higher in the rTMS plus rehabilitation group than the rehabilitation only group.

### Effect of treatment on serum BDNF, proBDNF and MMP-9 levels

**Fig 2:**
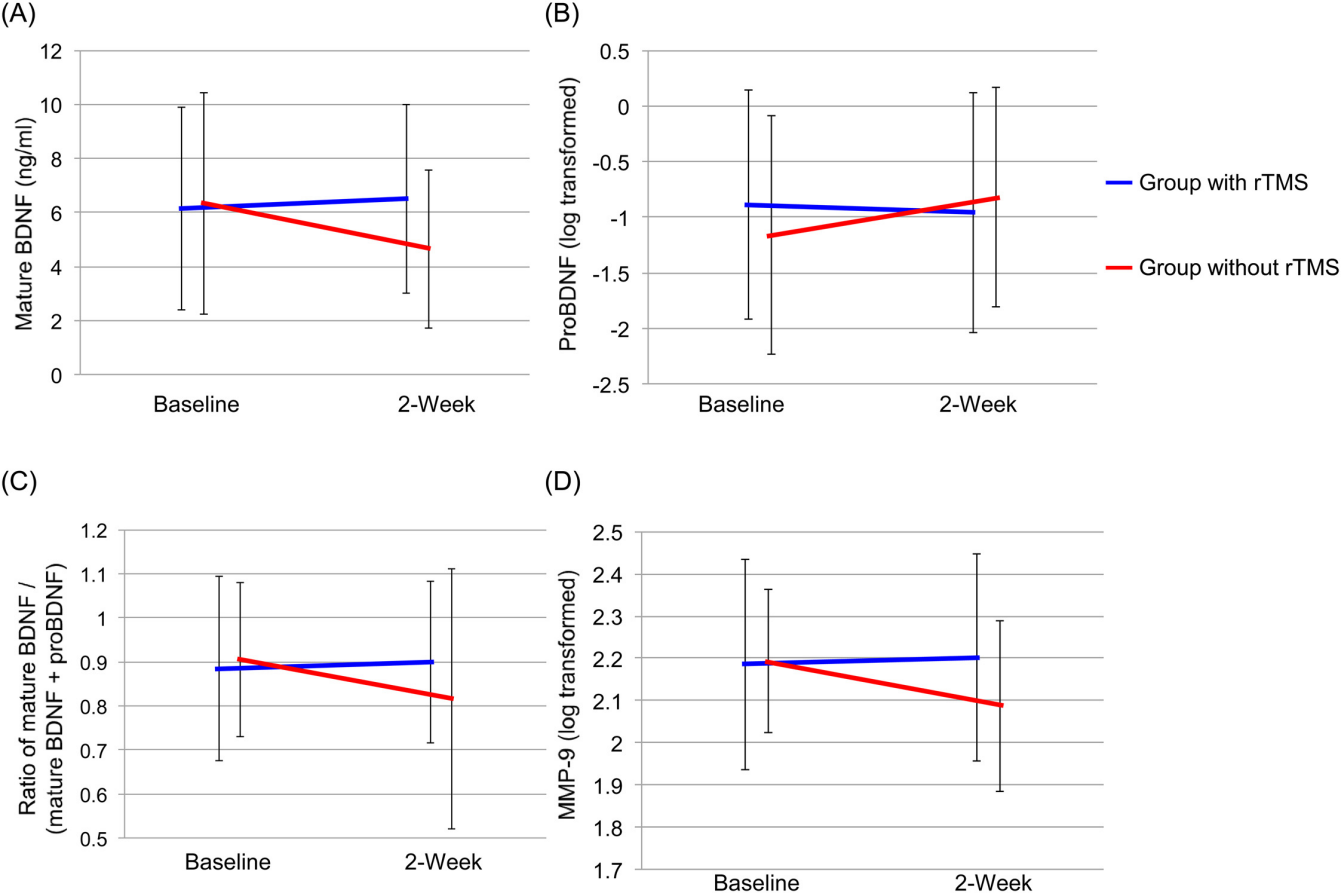
Effects of rehabilitation with and without 2-week rTMS on serum levels of biomarkers. (A) Mature BDNF: There was a significant interaction between group and time (F = 4.1, df = 1,93, p = 0.047). (B) ProBDNF: There was no significant interaction between group and time (F = 2.6, df = 1,65, p = 0.110). (C) BDNF/total BDNF (BDNF and proBDNF) ratio: There was a significant interaction between group and time (F = 6.4, df = 1,66, p = 0.014). (D) MMP-9: There was a significant interaction between group and time (F = 7.0, df = 1,93, p = 0.010).

Serum proBDNF levels were compared before and after treatment in 50 patients of the rTMS plus rehabilitation group and 26 patients before and 22 patients of the rehabilitation only group since serum levels of proBDNF in the other patients were below the minimum detectable concentration of the kit. Two-way repeated measures ANOVA of BDNF data showed a significant group-by-time interaction (group: F = 2.1, df = 1,93, p = 0.152, time: F = 1.7, df = 1,93, p = 0.193, interaction (group x time): F = 4.1, df = 1,93, p = 0.047) (Fig 2A). There were no significant effects and interaction in serum proBDNF levels (group: F = 0.2, df = 1,66, p = 0.685, time: F = 0.1, df = 1,66, p = 0.730, interaction (group x time): F = 2.6, df = 1,66, p = 0.110) (Fig 2B). Furthermore, two-way repeated measures ANOVA of the ratio of BDNF to total BDNF (BDNF plus proBDNF) showed a significant group-by-time interaction (group: F = 0.3, df = 1,66, p = 0.580, time: F = 2.6, df = 1,66, p = 0.109, interaction (group x time): F = 6.4, df = 1,66, p = 0.014) (Fig 2C).

Two-way repeated measures ANOVA of MMP-9 data showed a significant group-by-time interaction (group: F = 1.5, df = 1,93, p = 0.220, time: F = 3.9, df = 1,93, p = 0.053, interaction (group x time): F = 7.0, df = 1,93, p = 0.010) (Fig 2D).

### Correlations with clinical evaluation of upper limb motor function

**Fig 3:**
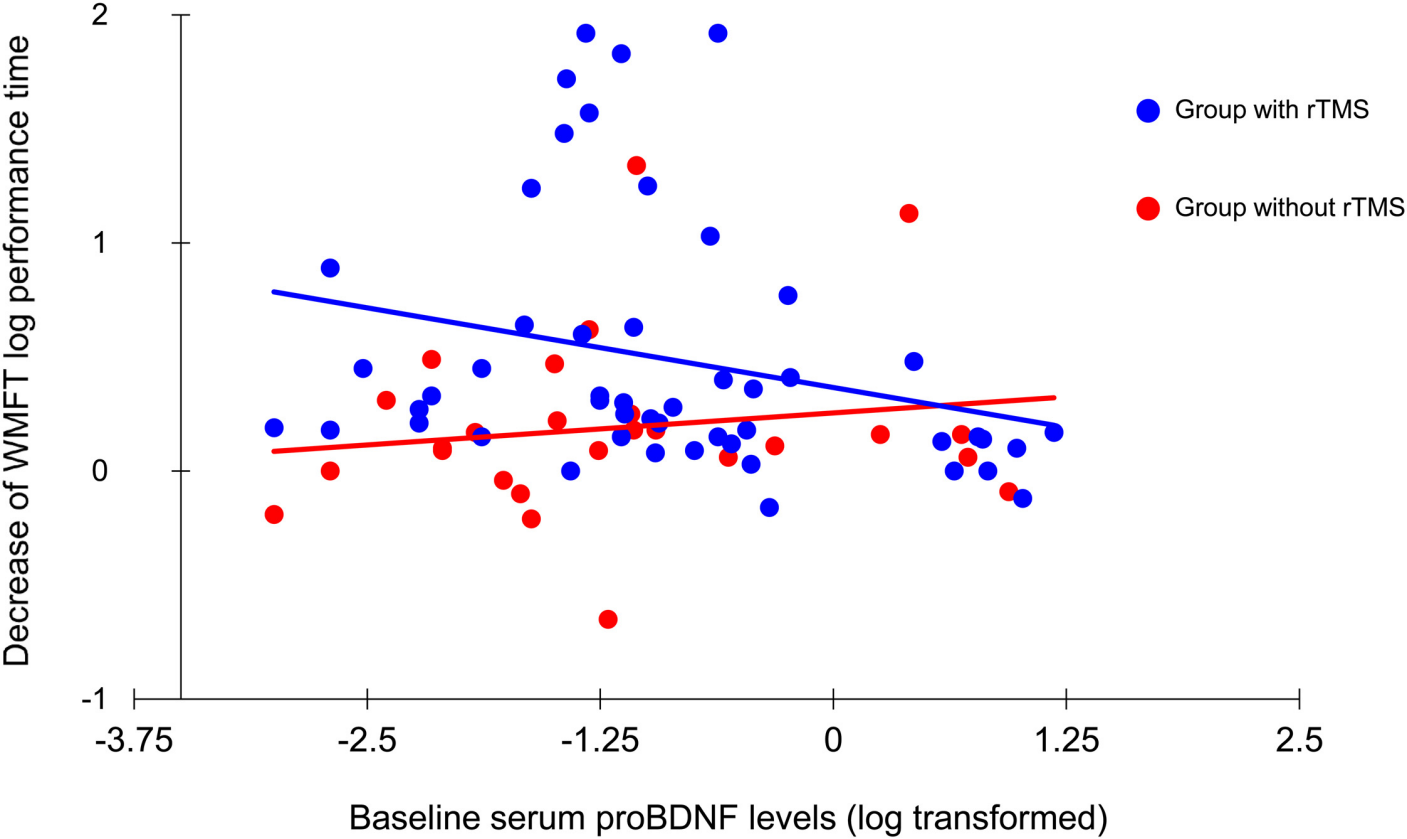
Correlation between baseline serum proBDNF levels and decrease in WMFT log performance time. There was a significant correlation between serum proBDNF levels and decrease in WMFT log performance time in patients treated with rehabilitation and rTMS (n = 50, ρ = -0.422, p = 0.002) but not in those treated with rehabilitation only.

Baseline BDNF and MMP-9 serum levels did not correlate with changes in FMA and WMFT log performance time in both treatment groups. Furthermore, proBDNF serum levels did not correlate with the increase in FMA score in both groups. Interestingly, there was a significant negative (ρ = -0.422, p = 0.002) correlation between proBDNF serum levels and decrease in WMFT log performance time in the rTMS plus rehabilitation group (n = 50), but not in the rehabilitation group (n = 26) (Fig 3).

### Effect of BDNF Val66Met polymorphism on improvement in motor function

**Fig 4:**
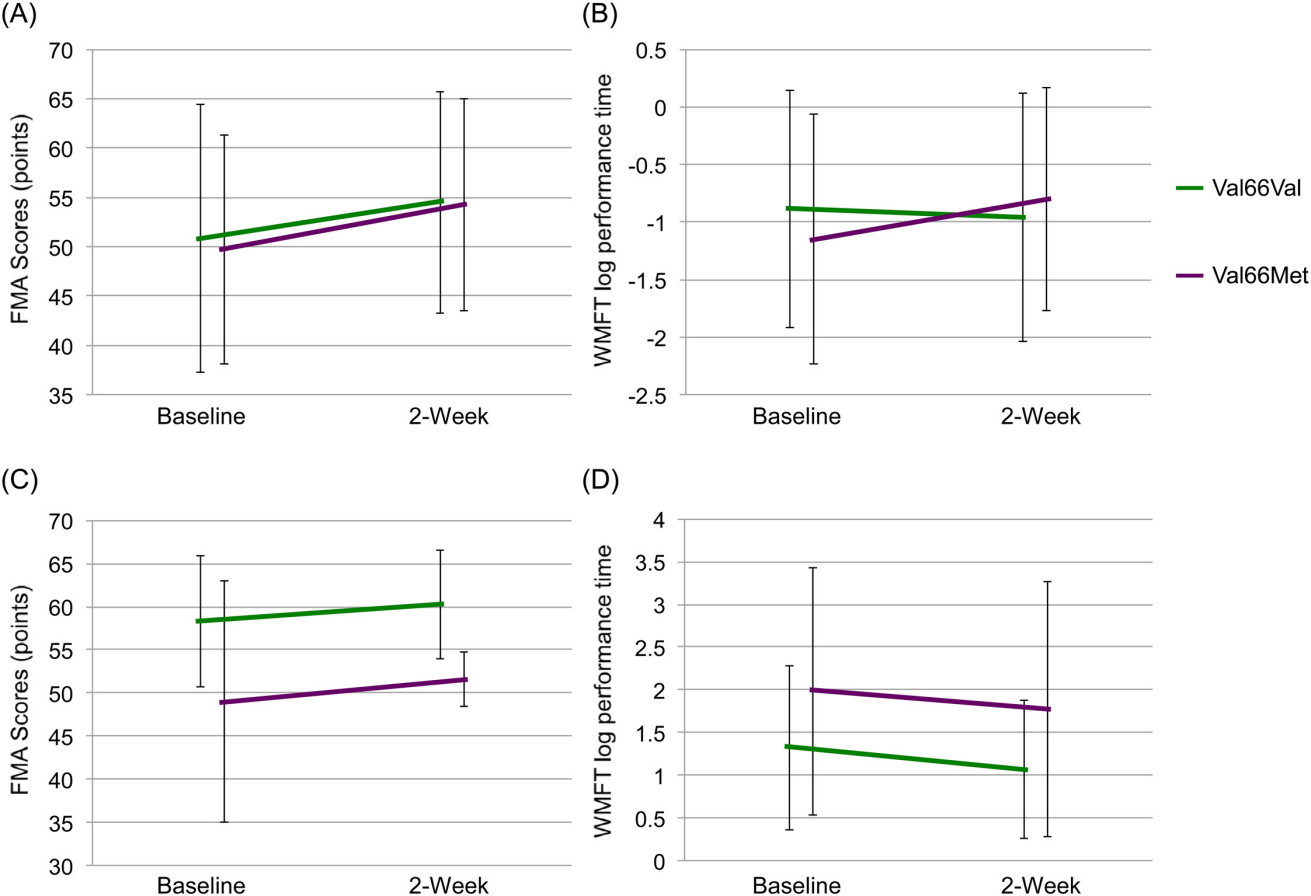
Effects of BDNF Val66Met gene polymorphism on FMA and WMFT log performance time. Effects of BDNF Val66Met gene polymorphism on (A) FMA and (B) WMFT log performance time in patients who received rehabilitation plus rTMS combination therapy. Effects of rehabilitation monotherapy on FMA (C) and WMFT log performance time (D) in Val66Val and Val66Met patients.

Based on the small number of patients with met allele, we divided the patients into two groups (val66val vs. val66met and met66met). Two-way repeated measures ANOVA of FMA data showed statistical significance in the rTMS plus rehabilitation group (gene: F = 0.05, df = 1,60, p = 0.828, time: F = 87.0, df = 1,60, p<0.001, interaction (gene x time): F = 1.0, df = 1,60, p = 0.333) (Fig 4A), as well as in the rehabilitation group (gene: F = 4.8, df = 1,31, p = 0.036, time: F = 21.0, df = 1,31, p<0.001, interaction (gene x time): F = 0.4, df = 1,31, p = 0.513) (Fig 4B).

Two-way repeated measures ANOVA of WMFT log performance time data showed statistical significance in the rTMS plus rehabilitation group (gene: F = 0.1, df = 1,60, p = 0.740, time: F = 45.3, df = 1,60, p<0.001, interaction (gene x time): F = 2.7, df = 1,60, p = 0.108) (Fig 4C), as well as in the rehabilitation monotherapy group (gene: F = 2.5, df = 1,31, p = 0.127, time: F = 7.2, df = 1,31, p = 0.012, interaction (gene x time): F = 0.1, df = 1,31, p = 0.798) (Fig 4D).

## Discussion

This is the first report to show that 2-week low-frequency rTMS increases serum levels of mature BDNF and MMP-9 in stroke patients. Given the key role of BDNF-TrkB signaling in neuroplasticity, it is likely that high serum levels of mature BDNF play at least some role in the beneficial actions of rTMS on motor function in the affected upper limb.

Accumulating evidence suggests that rTMS is effective treatment for poststroke hemiparesis [1]. Rehabilitation with low-frequency rTMS resulted in greater improvement of upper limb hemiparesis than rehabilitation without rTMS. In the present study, the rTMS plus rehabilitation group showed better improvement in FMA and WMFT than the rehabilitation alone group, as reported previously by Abo et al. [29].

The rTMS treatment is reported to result in significant increases in serum levels of total BDNF (mature BDNF + proBDNF) in patients with depression [11]. However, another study showed no such changes in serum levels of total BDNF after rTMS combined with rehabilitation [30]. In the above two studies, the BDNF ELISA kit did not recognize BDNF (mature form) and proBDNF [15]. One possibility for the discrepancy between the studies is the use of ELISA kit. In the present study, we found that serum levels of mature BDNF increased after 2-week rTMS plus rehabilitation, but decreased after 2-week rehabilitation only. Furthermore, serum levels of MMP-9 increased after 2-week rTMS plus rehabilitation whereas they decreased after 2-week rehabilitation only. Considering the role of MMP-9 in the conversion of proBDNF to mature BDNF, it is interesting to note the high levels of serum mature BDNF after 2-week rTMS.

Accumulating evidence indicates that both proBDNF and mature BDNF play important roles in various physiological functions, eliciting opposite effects via the p75^NTR^ and TrkB receptors, respectively [7,8,14]. These two parameters have not so far been measured accurately due to the limited specificity of the BDNF antibody. Luckily, the newly available ELISA Kit allowed us to measure separately the levels of both BDNF (mature form) and proBDNF [15]. Our results showed that rTMS plus rehabilitation slightly reduced proBDNF serum levels, whereas rehabilitation alone resulted in a slight increase in proBDNF serum levels. Based on the above changes, rehabilitation alone resulted in reduction in the ratio of mature BDNF/total BDNF, whereas it increased slightly in patients of the rTMS plus rehabilitation group. These findings suggest that low-frequency rTMS can activate the conversion of proBDNF to mature BDNF, and that such change enhances the effects of rehabilitation therapy on improvement of upper limb hemiparesis.

The results also showed a negative correlation between baseline serum proBDNF and improvement in WMFT, reflecting improvement in upper limb motor function. This preliminary result suggests that high baseline level of serum proBDNF could be used to predict poor improvement of motor function in the affected upper limbs, or alternatively, used as a biomarker of the response to rehabilitation in poststroke patients with hemiparesis. Further studies of large samples are needed to confirm these conclusions.

Several studies have reported that *BDNF* gene val66met polymorphism has a negative effect on the outcome of rTMS therapy [13,31]. However, the present results indicate that the outcome of rTMS therapy is not altered by this polymorphism, although we acknowledge the small number of patients included in the study. Nonetheless, our results demonstrate that rTMS combined with rehabilitation improves motor function in the affected limb after stroke irrespective of *BDNF* gene polymorphism. Whereas we did not determine the reason for the above differences between the two studies, we believe it relates to differences in the methodology; the rTMS sessions applied in the present study outnumbered those used in the previous study [13].

### Limitations

The present study has several limitations. First, it was not a randomized controlled trial, and rTMS treatment was directly applied to the patient. In addition, the sample size was too small to allow firm conclusions. Further randomized controlled studies of larger population are needed. Second, the sensitivity of the human proBDNF ELISA kits used in this study is less than ideal; in fact, serum levels of proBDNF in some patients were not detected, whereas they were detected in all subjects who participated in a Swedish study [32] and Israeli study [33], suggesting ethnic differences in serum proBDNF levels. The development of highly sensitive proBDNF ELISA kits is desirable for accurate measurement of proBDNF serum levels.

## Conclusions

Two-week low-frequency rTMS plus rehabilitation therapy resulted in greater improvement of upper limb motor function than rehabilitation alone, and this effect was mediated, at least in part, through rises in serum levels of mature BDNF and MMP-9 in poststroke patients. Mature BDNF, proBDNF, and MMP-9 could be potentially useful biomarkers of the response to therapy in poststroke patients with upper limb motor deficit.
